# Long-Term Prognosis Value of Paravalvular Leak and Patient–Prosthesis Mismatch following Transcatheter Aortic Valve Implantation: Insight from the France-TAVI Registry

**DOI:** 10.3390/jcm11206117

**Published:** 2022-10-17

**Authors:** Pierre Deharo, Lionel Leroux, Alexis Theron, Jérome Ferrara, Antoine Vaillier, Nicolas Jaussaud, Alizée Porto, Pierre Morera, Vlad Gariboldi, Bernard Iung, Thierry Lefevre, Philippe Commeau, Margaux Gouysse, Florence du Chayla, Nicolas Glatt, Guillaume Cayla, Herve Le Breton, Hakim Benamer, Sylvain Beurtheret, Jean Philippe Verhoye, Helene Eltchaninoff, Martine Gilard, Jean Philippe Collet, Nicolas Dumonteil, Frederic Collart, Thomas Modine, Thomas Cuisset

**Affiliations:** 1 Département de Cardiologie, CHU Timone, 13385 Marseille, France; 2 INSERM, Inra, C2VN, Aix Marseille La Timone University, 13005 Marseille, France; 3 Faculté de Médecine, Aix-Marseille Université, 13005 Marseille, France; 4 Département de Cardiologie, CHU Bordeaux, 33075 Bordeaux, France; 5 Département de Chirurgie Cardiaque, CHU Timone, 13005 Marseille, France; 6 AP-HP, Cardiology Department, Bichat Hospital, Université Paris Cité, INSERM 1148, 46 rue Henri Huchard, 75018 Paris, France; 7 Hopital Privé Jacques Cartier, 6 Av. Noyer Lambert, 91300 Massy, France; 8 Cardiologie Interventionnelle, Polyclinique Les Fleurs, Groupe ELSAN, 83190 Ollioules, France; 9 Clinityx, 78620 Acheres, France; 10 Department of Cardiology, CHU Nîmes, 30029 Nimes, France; 11 Service de Cardiologie, Hôpital Pontchaillou, Centre Hospitalier Universitaire de Rennes, 35033 Rennes, France; 12 Saint-Joseph Hospital, 13000 Marseille, France; 13 Rennes University Hospital, 35033 Rennes, France; 14 Department of Cardiology, Normandie University, UNIROUEN, U1096, CHU Rouen, 76000 Rouen, France; 15 Department of Cardiology, CHRU Brest, 29200 Brest, France; 16 Department of Cardiology, Sorbonne Université, INSERM UMRS_1166, Pitié Salpêtrière (AP-HP), 75000 Paris, France; 17 Groupe CardioVasculaire Interventionnel, Clinique Pasteur, 31300 Toulouse, France

**Keywords:** TAVI, mismatch, paravalvular leak

## Abstract

Background: Transcatheter aortic valve implantation (TAVI) is the preferred treatment for symptomatic severe aortic stenosis (AS) in a majority of patients across all surgical risks. Patients and methods: Paravalvular leak (PVL) and patient–prosthesis mismatch (PPM) are two frequent complications of TAVI. Therefore, based on the large France-TAVI registry, we planned to report the incidence of both complications following TAVI, evaluate their respective risk factors, and study their respective impacts on long-term clinical outcomes, including mortality. Results: We identified 47,494 patients in the database who underwent a TAVI in France between 1 January 2010 and 31 December 2019. Within this population, 17,742 patients had information regarding PPM status (5138 with moderate-to-severe PPM, 29.0%) and 20,878 had information regarding PVL (4056 with PVL ≥ 2, 19.4%). After adjustment, the risk factors for PVL ≥ 2 were a lower body mass index (BMI), a high baseline mean aortic gradient, a higher body surface area, a lower ejection fraction, a smaller diameter of TAVI, and a self-expandable TAVI device, while for moderate-to-severe PPM we identified a younger age, a lower BMI, a larger body surface area, a low aortic annulus area, a low ejection fraction, and a smaller diameter TAVI device (OR 0.85; 95% CI, 0.83–0.86) as predictors. At 6.5 years, PVL ≥ 2 was an independent predictor of mortality and was associated with higher mortality risk. PPM was not associated with increased risk of mortality. Conclusions: Our analysis from the France-TAVI registry showed that both moderate-to-severe PPM and PVL ≥ 2 continue to be frequently observed after the TAVI procedure. Different risk factors, mostly related to the patient’s anatomy and TAVI device selection, for both complications have been identified. Only PVL ≥ 2 was associated with higher mortality during follow-up.

## 1. Introduction

Transcatheter aortic valve implantation (TAVI) is considered to be the preferred treatment for symptomatic severe aortic stenosis (AS) in a majority of patients across all surgical risks [1]. Presently, TAVI has become the most frequent aortic valve replacement modality in developed countries, exceeding surgical aortic valve replacement [2]. Therefore, the continuous assessment of long-term results of this percutaneous AS treatment is critical.

Paravalvular leak (PVL) was initially identified as one of the most frequent complications following TAVI and has been associated with poor clinical outcomes, including death [3,4]. Consequently, a newer generation of TAVI devices have been designed to reduce the risk of PVL with the addition of an external skirt to the aortic bioprosthesis [5,6]. However, with those newer generations of TAVI devices being bulkier, patient–prosthesis mismatch (PPM) has emerged as a frequent echocardiographic finding following TAVI [7]. While it has been extensively studied in surgical aortic valve replacement, the long-term prognostic impact of PPM following TAVI continues to be debated with conflicting evidence [8,9]. The respective impact of these two “sub-optimal” results of TAVI have not been compared in a large dataset with a long follow-up.

Therefore, based on the large France-TAVI registry, we planned to report the incidence of both complications following TAVI, evaluate their respective risk factors, and study their respective impacts on long-term clinical outcomes, including mortality.

## 2. Methods

### 2.1. TAVI Registries

Designed as all-comer registries, FRANCE 2 and France-TAVI prospectively include patients undergoing TAVI for severe AS in France. The FRANCE 2 registry includes all implanted patients from January 2010 to January 2012, and the design was previously described [10,11]. The France-TAVI registry was launched in January 2013 and includes all patients who underwent TAVI in 48 of the 50 active TAVI centers in France. France-TAVI is an initiative of the working group of interventional cardiology of the French Society of Cardiology with the participation of the French Society of Thoracic and Cardiovascular Surgery.

Both these large, national, multicenter, prospective registries were designed to provide baseline characteristics of the patients as well as TAVI procedural aspects. As previously described, the decision to perform TAVI and the choice of access and type of device were made based on an assessment by a multidisciplinary heart team [10,11,12]. Procedures and postprocedural management were performed in accordance with each site’s routine protocol. A 30-day follow-up was recommended in the case report form and was performed either on site or by telephone contact with the patient and the patient’s physician, depending on each site’s protocol. The dataset was collected using a dedicated web-based interface managed by the French Society of Cardiology, which implements regular data quality checks, including range checks and assessments of internal consistency.

All patients provided written informed consent for the anonymous processing of their data, and the institutional review board of the French Ministry of Health approved the registry. All data are the property of the French Society of Cardiology and were collected with the participation of the French Society of Thoracic and Cardiovascular Surgery.

In France, the single-payer national health data system (SNDS) provides access to data on national health insurance payments. It covers almost 99% of the French population, or more than 66 million people, making it one of the world’s largest continuous homogeneous health-claim databases [13]. The SNDS was created in 2015 by a merger of the SNIIRAM (Système National d’Information Inter-régime de l’Assurance Maladie) (national anonymous claims database), PMSI (Programme de Médicalisation des Systèmes d’Information) (hospital activity database), national diagnosis-related group (DRG) database, and CepiDC (Centred’Epidémiologie des Causes Médicales de Décès) (national registry of causes of death).

A linking algorithm was developed to match patients in TAVI registries with data from the SNDS. The linkage process employed a probabilistic approach based on matching SNDS data as closely as possible to the profiles in the registry databases [13]. SNDS entries with failure of probabilistic linkage or false patient data (wrong date of birth, wrong admission date, multiple dates of death, or duplicate data) were excluded. All data were analyzed anonymously. Each individual record in the SNDS was randomly assigned a numerical identity that did not include any information regarding the patient or center identities. This number was used in the present analysis, with no reverse identification possible.

Since all patient data were extracted from registries, informed consent and ethical clearance had already been obtained, and a specific authorization from the national data protection commission (CNIL) was received for the SNDS linkage.

### 2.2. Data Collection

Most baseline and procedural characteristics and in-hospital results as well as 30-day echocardiographic follow-up data were extracted from the FRANCE 2 and France-TAVI registries, while all clinical events occurring after the indexed hospitalization discharge were extracted from the SNDS database.

All ICD-10, medical procedure, and ATC codes used to identify variables from the SNDS are detailed in Supplementary Table S1.

### 2.3. Study Design

#### 2.3.1. Study Groups and PPM and PVL Definitions

For the purposes of this analysis, all patients included from 1 January 2010 to 31 December 2019 in the FRANCE 2 and France-TAVI databases were screened.

Patients were excluded from this analysis if they had a follow-up <30 days after TAVI, if they underwent TAVI with a device other than an Edwards Sapien (Sapien, Sapien XT or Sapiens 3) or a Medtronic CoreValve (CoreValve, Evolut R, or Evolut Pro), in case of a valve-in-valve TAVI, in case of a TAVI for pure aortic regurgitation, and in case of missing data needed to assess PVL and/or PPM. The number of patients with TAVI devices other than Edwards Sapiens or Medtronic CoreValve was very low.

Postprocedural TTE was intended to be performed on day 2 after the procedure and was performed, at the latest, before hospital discharge and at day 30. Mitral and aortic regurgitation were assessed using a colorflow Doppler signal and graded in five groups as none or trivial (=0/4), mild (=1/4), mild-to-moderate (=2/4), moderate-to-severe (=3/4), or severe (=4/4). Native and post-TAVR ARs were evaluated according to the European Association of Echocardiography guidelines [14] and the American Society of Echocardiography recommendations [15] by the use of a multiparametric and integrative approach rather than a single measurement. In the case of post-TAVR ARs, because they are often paravalvular, the evaluation relied more heavily on the circumferential extent of the paravalvular jet(s), as evaluated just below the bioprosthesis on the short-axis view, than on the other parameters.

None, mild, mild-to-moderate, moderate-to-severe, and severe post-TAVRs were defined according to American Society of Echocardiography guidelines [15] with the following adaptation that, similar to the European Association of Echocardiography proposal for the evaluation of ARs of the native valves, [15] moderate post-TAVR ARs were subdivided in mild-to-moderate and moderate-to-severe ARs. When several AR jets were present, AR was expressed as an overall grade, unless otherwise stated. A valvular regurgitation ≥ 2 was considered significant.

The effective orifice area (EOA) was calculated according to the continuity equation. The indexed EOA (iEOA) was calculated as the EOA divided by the body surface area (BSA). Moderate PPM was defined by 0.65 ≤ iEOA ≤ 0.85 cm^2^/m^2^ (0.55 ≤ iEOA ≤ 0.70 cm^2^/m^2^ if BMI ≥ 30 kg/m^2^), and severe PPM was defined by an iEAO < 0.65 cm^2^/m^2^ (≤0.55 if BMI ≥ 30 kg/m^2^) [16].

We divided the whole population into two groups according to the PVL grade (PVL < 2 and PVL ≥ 2) and into two groups according to PPM (moderate-to-severe PPM versus no PPM).

TAVI devices were divided into two groups: balloon-expandable (BE) Edwards Sapien (Edwards Lifesciences Inc., Irvine, CA, USA) and self-expandable (SE) Medtronic CoreValve (Medtronic Inc., Minneapolis, MN, USA).

#### 2.3.2. Endpoints

The primary endpoint of the study was death from any cause, which was extracted from the SNDS as the date of death, during follow-up according to PVL ≥ 2 or moderate-to-severe PPM presence.

Secondary endpoints included:

– The incidence and identification of the risk factors for moderate-to-severe PPM and PVL ≥ 2 after TAVI;
– Rehospitalization for heart failure, stroke, aortic valve reintervention, pacemaker implantation at 30 days, or cardiac arrhythmia during follow-up;
– Predictors of all-cause mortality.

#### 2.3.3. Statistical Analysis

Absolute numbers, percentages, and means ± SD or median (interquartile range (IQR)) were computed to describe the populations. Comparisons between groups used the Mann–Whitney test for continuous variables, as all of them were not normally distributed, and the chi-square test for categorical variables.

Multivariate logistic regressions were performed to identify risk factors for PPM and PVL ≥ 2 after TAVI. Clinically relevant candidate variables with *p*-values < 0.2 in the univariate analysis were included in the multivariate model. Variables with more than 15% missing data were excluded.

The Kaplan–Meier method was used to estimate the 6.5-year all-cause mortality rate, while cumulative incidence rates for HF rehospitalization, stroke, aortic valve reintervention, pacemaker implantation, and cardiac arrhythmia were analyzed with the Kalbfleisch and Prentice method to account for all-cause death as competing risks. Comparisons between groups were assessed with the log-rank test for all-cause mortality and with the gray test for the other clinical outcomes.

To evaluate the impact of PPM and PVL ≥ 2 on 6.5-year all-cause mortality, multivariate Cox regression models adjusted to baseline and procedural (including in-hospital complications) characteristics were used. Clinically relevant and significant (*p*-value < 0.2) variables in the univariate analysis were introduced into multivariate models, while variables with more than 15% missing data were excluded. The moderate-to-severe PPM variable was forced into the multivariate analysis, as it was not significant in the univariate analysis. Proportional hazard assumptions of each factor were checked using a test and graphical diagnosis based on Schoenfeld residual plots.

The Python stats library was used for data analysis, and the Python matplotlib library was used to plot graphs. The Python packages lifeline and statsmodels were employed for the Cox and logistic regression models. The Kalbfleisch and Prentice models were performed using the R library cmprsk in the rpy2 interface.

## 3. Results

### 3.1. Baseline Characteristics

**Figure 1:**
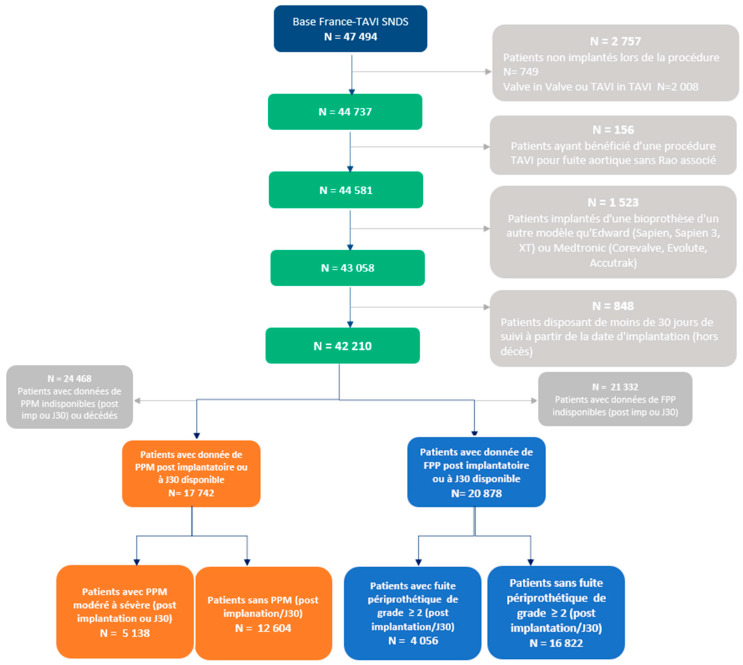
Flow chart.

We identified 47,494 patients in the database who underwent a TAVI in France between 1 January 2010 and 31 December 2019. After the exclusion of valve-in-valve TAVI (*n* = 2008), TAVI with pure aortic regurgitation as indication (*n* = 156), TAVI with a valve than an Edwards or Medtronic (*n* = 1523), and patients with follow-up at less than 30 days (*n* = 848), we obtained a study population of 42,210 patients who underwent TAVI for severe AS (Figure 1).

### 3.2. Incidence and Risk Factors for PPM and PVL

Within this population, 17,742 patients had complete information regarding PPM status (5138 with moderate-to-severe PPM, 29.0 %) and 20,878 had complete information regarding PVL (4056 with PVL ≥ 2, 19.4%) (Figure 1). The baseline characteristics of the PPM vs. no PPM cohorts and the PVL vs. no PVL cohorts are presented in Table 1 and Table 2.

Of note, patients with moderate-to-severe PPM, as compared to those without, were younger and more often female with a higher BMI. The baseline effective aortic area in echocardiography was lower (0.68 ± 0.2 vs. 0.71 ± 0.2, *p* < 0.01), and those patients presented more often with severe pulmonary hypertension (12% vs. 10%, *p* < 0.01).

Patients with PVL ≥ 2 were older and had lower BMI than those with PVL < 2. They had less cardiovascular risk factors and history of coronary or lung disease at baseline. At baseline, higher TTE gradients were reported in those patients.

Multivariate adjustment results are provided in Table 3 and Table 4.

After adjustment, the risk factors for PVL ≥2 were a lower body mass index (OR 0.962; 95% CI, 0.951–0.973), a higher baseline mean aortic gradient (OR 1.009; 95% CI, 1.007–1.011), a higher body surface area (OR 1.357; 95% CI, 1.038–1.774), a lower baseline ejection fraction (OR 0.995; 95% CI, 0.992–0.998), a smaller diameter size of the TAVI valve (OR 0.972; 95% CI, 0.959–0.986), and the use of SEV (BEV vs. SEV OR 0.509; 95% CI, 0.469–0.552).

Regarding moderate-to-severe PPM, we identified a younger age (OR 0.992; 95% CI, 0.987–0.997), BMI (OR 0.96; 95% CI, 0.95–0.97), a higher body surface area (OR 8.88; 95%CI 6.72–11.74), a low aortic annulus area (OR 0.35; 95% CI, 0.27–0.46), a lower baseline ejection fraction (OR 0.985; 95% CI, 0.982–0.988), and a smaller TAVI device diameter (OR 0.85; 95% CI, 0.83–0.86) as predictors of moderate-to-severe PPM.

### 3.3. Outcomes according to PVL and PPM

#### 3.3.1. Impact of PPM on Clinical Outcomes

**Figure 2:**
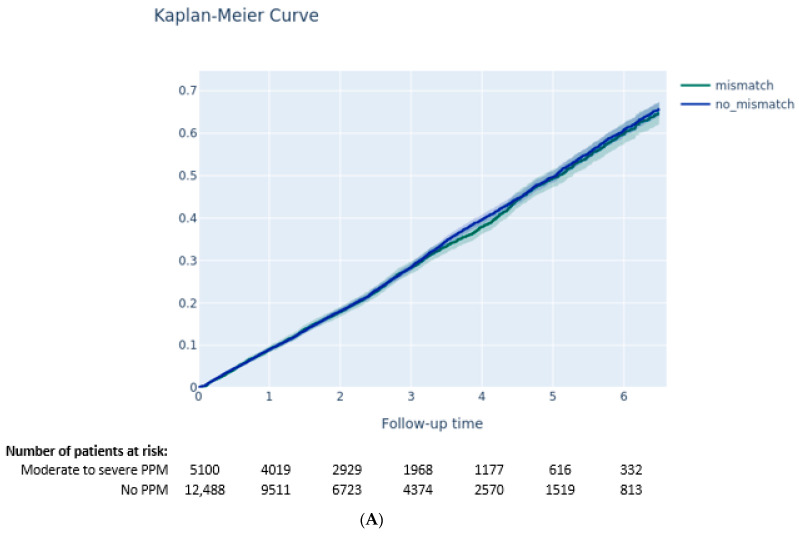

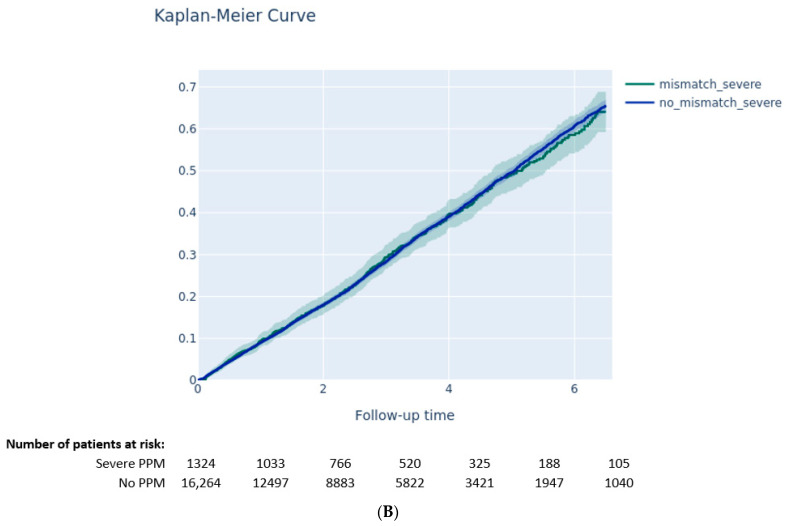
(**A**) Survival according to the presence of moderate-to-severe patient–prosthesis mismatch (Kaplan–Meier curve). (**B**) Survival according to the presence of severe patient–prosthesis mismatch (Kaplan–Meier curve).

**Figure 3:**
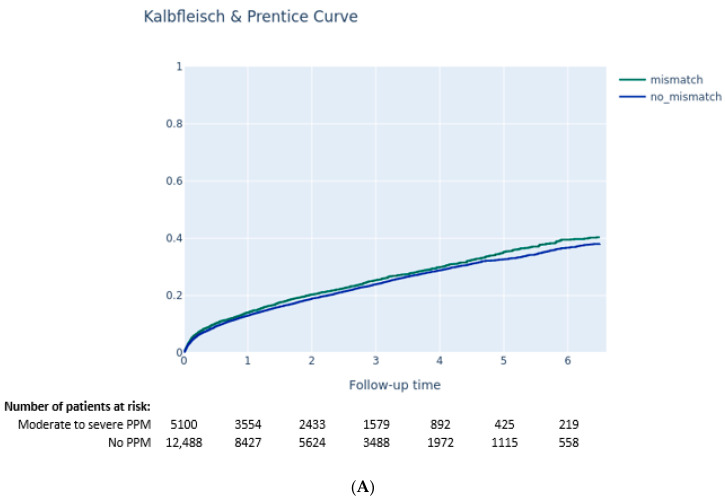

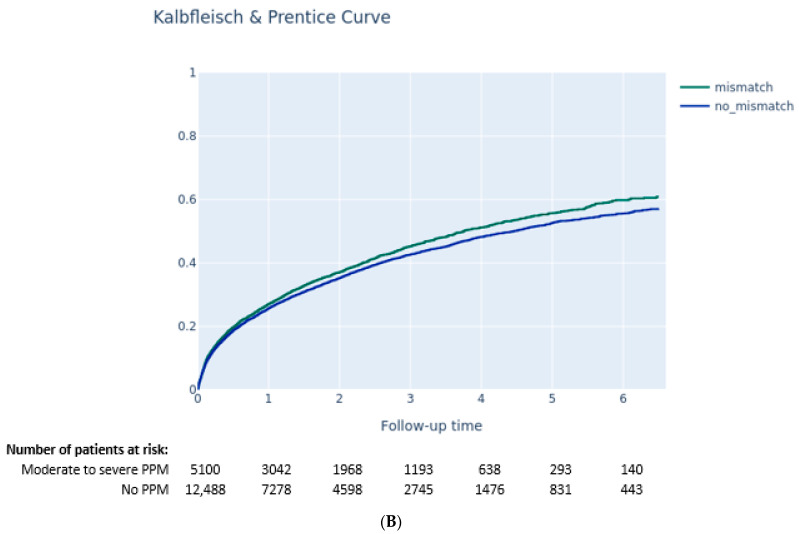
(**A**) Cumulative incidence of occurrence of first hospitalization for heart failure according to the presence of moderate-to-severe patient–prosthesis mismatch (Kalbfleisch and Prentice curve). (**B**) Cumulative incidence of occurrence of arrhythmia according to the presence of moderate-to-severe patient–prosthesis mismatch (Kalbfleisch and Prentice curve).

At 6.5 years following TAVI, neither moderate-to-severe PPM (64.7% vs. 65.7, *p* = 0.4) nor severe PPM (64.1% vs. 65.5% mortality, *p* = 0.8) were associated with increased risk of all-cause mortality (Figure 2A,B). Moderate-to-severe PPM was associated with higher rates of rehospitalization for heart failure (40.4% vs. 38.1%, *p* = 0.03) (Figure 3A). Arrhythmias were reported more often in cases of moderate-to-severe PPM (61.0% vs. 57.1% *p* = 0.0013) (Figure 3B) and severe PPM (63.8% vs. 57.7% *p* = 0.003).

Moderate-to-severe PPM was not associated with differences in terms of aortic reintervention (2.7% vs. 2.5%, *p* = 0.55), pacemaker implantation (8.5% vs. 8.1%, *p* = 0.6), or stroke (9.50% vs. 9.49%, *p* = 0.6).

#### 3.3.2. Impact of PVL on Clinical Outcomes

**Figure 4:**
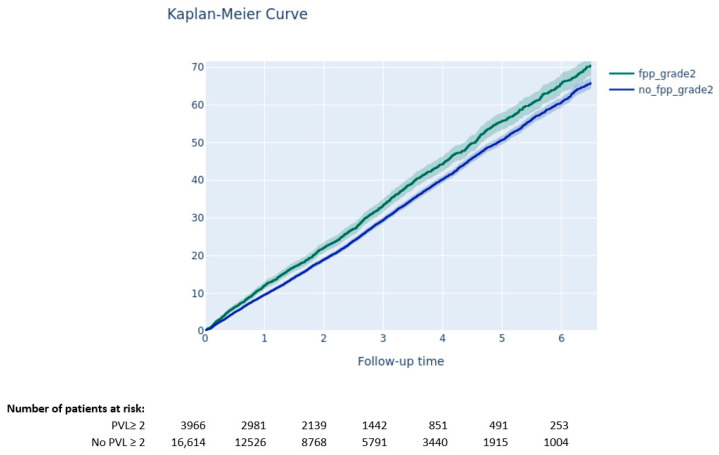
Survival according to the presence of grade ≥ 2 paravalvular leak (Kaplan–Meier curve).

At 6.5 years following TAVI, PVL ≥ 2 was associated with higher mortality risk during follow-up (70.5% vs. 65.8%, *p* < 0.001) (Figure 4).

**Figure 5:**
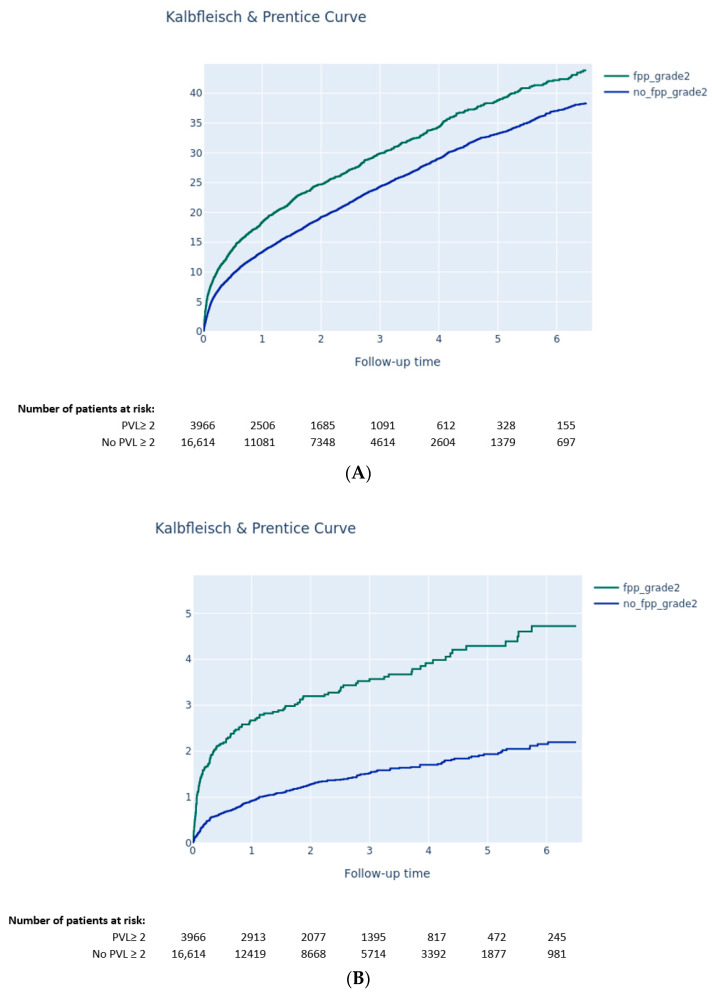

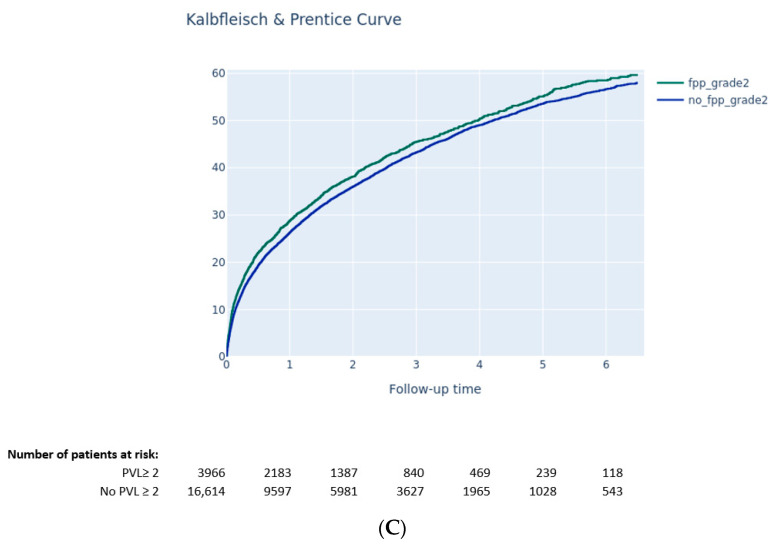
(**A**) Cumulative incidence of occurrence of first hospitalization for heart failure according to the presence of grade ≥ 2 paravalvular leak (Kalbfleisch and Prentice curve). (**B**) Cumulative incidence of occurrence of aortic valve reintervention according to the presence of grade ≥ 2 paravalvular leak (Kalbfleisch and Prentice curve). (**C**) Cumulative incidence of arrythmia according to the presence of grade ≥ 2 paravalvular leak (Kalbfleisch and Prentice curve).

Moreover, PVL was associated with higher rates of rehospitalization for heart failure (43.8% vs. 38.3%, *p* < 0.001) (Figure 5A), higher risk of aortic reintervention (4.7% vs. 2.2%, *p* < 0.001) (Figure 5B), and higher rates of arrythmia PM implantation (59.6% vs. 58.0%, *p* = 0.005) (Figure 5C). No associations between PVL and PM implantation (9.4% vs. 8.5%, *p* = 0.19) or incidence of stroke (8.1% vs. 9.3%, *p* = 0.05) were reported.

A multivariate adjustment of the predictors of all-cause mortality was performed for PPM and PVL (Table 5 and Table 6).

In the multivariate analysis, the predictors of all-cause mortality in the PPM cohort were an older age (HR 1.01), a lower BMI (HR 0.99), a higher Euroscore (HR 1.01), a history of chronic obstructive pulmonary disease (HR 1.19), NYHA classes 3 and 4 (HR 1.23), a higher baseline mean gradient (HR 0.99), a greater size of the TAVI device (HR 1.02), major bleeding (HR 1.23), and the length of hospital stay (HR 1.02).

In the multivariate analysis, the predictors of all-cause mortality in the PVL cohort were an older age (HR 1.01), a higher Euroscore (HR 1.01), NYHA classes 3 and 4 (HR 1.22), BMI (HR 0.99), a history of chronic obstructive pulmonary disease (HR 1.26), a higher baseline mean gradient (HR 0.99), a greater diameter of TAVI device (HR 1.02), PVL ≥ 2 (HR 1.16), major bleeding (HR 1.18), and the length of hospital stay (HR 1.02).

## 4. Discussion

Our main results could be summarized as follows:

– Moderate-to-severe PPM and PVL ≥ 2 were reported in 29.0% and 19.4%, respectively, of TAVI patients in France between 2010 and 2019.
– The main risk factors for moderate-to-severe PPM are a younger age, a lower BMI, a higher body surface area, a lower ejection fraction, a smaller aortic annulus, and a smaller diameter of TAVI device, while the main risk factors for PVL ≥ 2 are higher aortic gradients pre-TAVI, a lower BMI, a higher body surface area, a lower ejection fraction, a smaller diameter of TAVI, and the use of an SEV TAVI.
– Moderate-to-severe PPM was not associated with a higher risk of long-term death, while PVL ≥ 2 after TAVI was associated with higher mortality.

### 4.1. Background

The development of TAVI has offered a life-saving option for a paramount number of increasingly younger patients suffering from severe symptomatic AS. With the extension of TAVI indications, PVL was quickly identified as the Achilles’ heel of first-generation TAVI devices. Indeed, PVL was initially frequently observed, with up to 22% of patients presenting moderate or severe PVL. Therefore, the latest generation of TAVI device technology aimed to reduce the occurrence of PVL ≥ 2. Despite this significant improvement, the rates of PVL ≥ 2 remain more frequent after TAVI in comparison with SAVR [17,18].

### 4.2. Physiopathology and PPM and PVL Risk Factors

Severe AS is associated with left ventricle remodeling, stiffer ventricles, and reduced diastolic compliance. In the case of PVL, the ventricle may struggle to accommodate a sudden increase in LV pre- and afterload (owing to an increase in stroke volume), especially if onset occurs after AS correction. This volume overload translates into an increase in left atrial pressure, with a subsequent rise in pulmonary pressure. Consequently, PVL ≥ 2 is associated with worse outcomes after TAVI and increased risk of long-term mortality, as emphasized by our results [3,4,19]. This impact of PVL after TAVI may vary according to the presence of initial aortic regurgitation. However, we were not able to analyze this parameter.

PPM was initially defined as an effective prosthesis area lower than that of the normal human valve [20]. PPM occurs when the effective orifice area of the prosthetic valve is too small in relation to the patient’s body size, thus resulting in high procedural residual gradients despite normal prosthetic valve function. In the case of aortic valve replacement, the incidence of PPM was lower after the first and newer generations of TAVI compared to surgical AVR [21,22]. However, due to the addition of an external skirt to both BEV and SEV to limit the risk of PVL, the last generation of BEV has been associated with higher rates of PPM compared to SAVR [5].

PPM is associated with higher post-TAVI gradients and lower LV mass regression and diastolic dysfunction correction. The hemodynamic consequences of PPM become especially relevant in patients with factors that exacerbate residual LV afterload (such as severe LV hypertrophy in the case of AS) or in vulnerable patients (mainly younger age, severe MR, and low EF).

Therefore, PPM after TAVI has become a matter of concern and has been associated with increased risk of rehospitalization for HF and potentially death [8,22,23]. Moreover, a relation between the occurrence of PPM and the risk of early degeneration of the TAVI bioprosthesis has been hypothesized. In our cohort, PPM was not associated with a higher risk of aortic reinterventions, while rehospitalization for heart failure was more frequent in the case of PPM. No relation between PPM and long-term mortality was shown in our large cohort.

To prevent the occurrence of one or both of those long-term complications following TAVI, the identification of risk factors seems to be critical. Several predictors of PPM have been identified in the literature, including a small TAVI valve prosthesis, the VIV procedure, a larger BSA, a lower LV ejection fraction, being female, younger age, atrial fibrillation, a larger BMI, a higher aortic valve mean gradient, a prior CABG, and severe mitral or tricuspid regurgitation [8,21,22]. Our analysis confirmed that PPM was more prevalent in the case of high BMI, low ejection fraction, and small aortic annulus.

On the other hand, annular calcifications remain the more frequent factor associated with PVL [24]. In our cohort, higher pre-TAVI gradients (likely related to severely calcified AS) and the type of TAVI device were associated with PVL ≥ 2.

The accurate choice of valve design and the experience of TAVI operators improved significantly over time. However, despite these improvements, both complications remained frequent in our analysis. Indeed, at the French level, in an unselected population we observed 17% PVL ≥ 2 after TAVI.

Overall, our results are in favor of a stronger impact of PVL ≥ 2 compared to PPM. In fact, only PVL ≥ 2 was associated with long-term mortality. However, both complications were associated with a higher risk of rehospitalization for heart failure, and consequently we should aim to reduce the incidences of PPM and PVL in combination.

### 4.3. BEV and SEV

Since their introduction, comparisons between BEV and SEV devices in TAVI have always been matter of discussion. Those two different TAVI technologies offer specific advantages and disadvantages. Small randomized studies [25] and large registries [4,12,19,26] have suggested that PVL ≥ 2 was more frequent with SEV. Previous nonrandomized data from France-TAVI showed that SEV was associated with higher mortality compared to BEV, and a relation with higher PVL ≥ 2 has been hypothesized [19]. Due to the supra-annular design of the Evolut platform (SEV), the rates of PPM were reported lower than those observed in BEV [9]. In our large cohort, BEVs were associated with higher rates of moderate-to-severe PPM only in the univariate analysis, while we did not report a significant impact of TAVI technology on the risk of moderate-to-severe PPM in the multivariate analysis.

Therefore, PVL could be reduced by selecting a BEV in the case of a heavily calcified aortic annulus (to balance with the risk of aortic annulus rupture). PPM risk factors are mostly related to patient anatomy (BMI and annulus area), and it is likely that, in the case of identified high risk of PPM and likely poor clinical tolerance, SEV could be preferred and would achieve lower gradients.

### 4.4. Limitations

This is an observational registry study and has the inherent limitations associated with retrospective analyses, including residual measured and unmeasured confounding. However, this is a very large study, with all commercial TAVI procedures performed in France in a recent time frame. Second, a certain level of underreporting or missing echocardiographic data could exist, even if most of the relevant events were prospectively reported by the investigators in the course of the clinical follow-up or derived from an ad hoc analysis. Third, the relation between the diameter size of TAVI and the increased risk of mortality in the PVL cohort has not been explained.

## 5. Conclusions

In conclusion, our analysis from the France-TAVI registry showed that both moderate-to-severe PPM and PVL ≥ 2 continue to be frequently observed after the TAVI procedure. Different risk factors, mostly related to patient anatomy and TAVI device selection have been identified for both complications. While both complications were associated with a higher risk of rehospitalization for heart failure, only PVL ≥ 2 was associated with higher mortality during follow-up. Therefore, based on the identification of risk factors, the individualization of the TAVI device choice for every single patient should particularly aim to reduce the risk of both PVL and PPM.

## Figures and Tables

**Table 1:** Baseline and postimplantation characteristics (PPM cohort).

	All Patients	Moderate-to-Severe Mismatch (*n* = 5138)	No Moderate-to-Severe Mismatch (*n* = 12,604)	*p*
Duration of follow-up (days since the date of procedure)	816 (391.3–1360.8)	867 (427–1414)	795 (379–1341)	<0.001
Demography				
Age (years)	82.6 ± 6.8	82.0 ± 7.3	82.9 ± 6.6	<0.001
Female	8686 (49%)	2577 (50%)	6109 (48%)	0.04
Male	9056 (51%)	2561 (50%)	6495 (52%)	
BMI	26.2 (23.4–29.6)	26.6 (23.9–29.3)	26 (23.1–29.8)	<0.001
Body surface (m^2^)	1.8 (1.65–1.94)	1.82 (1.69–1.97)	1.78 (1.63–1.94)	<0.001
Indicators at inclusion				
NYHA III/IV	10,335 (62%)	3020 (63%)	7315 (62%)	0.14
Euroscore 2	3.72 (2.24–6.15)	3.86 (2.24–6.79)	3.69 (2.24–6)	<0.001
Logistic Euroscore	13.57 (8.9–21.43)	13.83 (8.78–21.69)	13.57 (8.97–21.3)	0.28
History and comorbidities				
Dyslipidemia	5487 (31%)	1624 (32%)	3863 (31%)	0.2
Hypertension	13,320 (75%)	3847 (75%)	9473 (75%)	0.71
CCS Class IV angina	549 (4%)	138 (3%)	411 (4%)	0.05
Coronary angioplasty	4661 (30%)	1316 (29%)	3345 (30%)	0.17
Coronary bypass	1604 (9%)	502 (10%)	1102 (9%)	0.04
Diabetes	4624 (26%)	1371 (27%)	3253 (26%)	0.21
Myocardial Infarction < 90 days	244 (2%)	86 (2%)	158 (1%)	0.04
History of stroke	1951 (11%)	564 (11%)	1387 (11%)	0.99
Chronic renal failure	7702 (50%)	2239 (50%)	5463 (50%)	0.23
Dialysis	309 (2%)	80 (2%)	229 (2%)	0.3
Creatinine (µmol/L)	92 (74–118)	93 (75–119.85)	91 (74–117)	<0.001
Chronic obstructive pulmonary disease	3426 (19%)	996 (19%)	2430 (19%)	0.9
Pacemaker	1961 (13%)	548 (12%)	1413 (13%)	0.3
Peripheral arterial disease	3793 (24%)	1112 (24%)	2681 (23%)	0.8
Preimplantation examination				
Severe pulmonary hypertension (>60 mmHg)	1452 (11%)	472 (12%)	980 (10%)	<0.001
Aortic valve area (cm^2^)	0.7 (0.58–0.8)	0.7 (0.55–0.8)	0.7 (0.6–0.82)	<0.001
Mean gradient (mmHg)	47 (40–57)	47 (39–57)	47 (40–56)	0.24
Ejection fraction (%)	60 (50–65)	60 (45–65)	60 (50–65)	<0.001
Aortic annulus diameter (mm)	23.5 (22–25.7)	23 (22–25.4)	23.6 (22–25.9)	<0.001
Aortic regurgitation ≥ 2	2695 (18%)	863 (20%)	1832 (18%)	<0.001
Mitral regurgitation ≥ 2	3345 (23%)	1011 (23%)	2334 (22%)	0.2
Coronary stenosis (>50%)	6901 (41%)	1952 (41%)	4949 (42%)	0.4
Procedure				
Programmed predilatation	4464 (37%)	1302 (36%)	3162 (37%)	0.5
Number of valves implanted > 1	200 (1%)	51 (1%)	149 (1%)	0.3
Access (iliofemoral)	15,329 (87%)	4410 (86%)	10,919 (87%)	0.15
Valve type				<0.001
SEV	5638 (32%)	1308 (25%)	4330 (34%)	
Corevalve and Corevalve Evolut	2229 (12%)	619 (12%)	1601 (12%)	
Corevalve Evolut Pro	897 (5%)	138 (3%)	759 (6%)	
Corevalve Evolut R	2512 (14%)	542 (11%)	1970 (16%)	
BEV	12,104 (68%)	3830 (75%)	8274 (66%)	
Sapien	1650 (9%)	497 (10%)	1153 (9%)	
Sapien 3	8971 (51%)	2931 (57%)	6040 (48%)	
Sapien XT	1483 (8%)	402 (8%)	1081 (9%)	
Diameter of the prosthesis (mm)	26 (23–29)	26 (23–26)	26 (26–29)	<0.001
Postimplant examination				
Ejection fraction (%)	60 (50–65)	60 (50–65)	60 (52–65)	<0.001
Mean gradient (mmHg)	10 (7–13)	12 (9–15)	9 (6.7–12)	<0.001
Aortic valve area (cm^2^)	1.78 (1.47–2.1)	1.31 (1.17–1.5)	1.9 (1.7–2.29)	<0.001
Indexed aortic valve area (cm^2^/m^2^)	0.99 (0.81–1.2)	0.74 (0.64–0.83)	1.08 (0.94–1.28)	<0.001
Mitral regurgitation ≥2	2361 (16%)	748 (17%)	1613 (16%)	0.02
Severe pulmonary hypertension (>60 mmHg)	638 (5%)	217 (6%)	421 (5%)	0.02
Events during hospitalization				
Pacemaker	2386 (15%)	640 (13%)	1746 (15%)	0.01
Infection	596 (4%)	182 (4%)	414 (4%)	0.4
ST+ infarction	22 (0%)	<11 (<0.2%) *	13 (0%)	0.3
Stroke	272 (2%)	78 (2%)	194 (2%)	0.95
Major bleeding	889 (5%)	242 (5%)	647 (6%)	0.23
Death	154 (1%)	38 (1%)	116 (1%)	0.31
Duration of index hospitalization (days)	8 (6–13)	8 (6–13)	8 (6–12)	<0.001
Grade ≥ 2 periprosthetic aortic leak	1055 (16%)	317 (16%)	738 (16%)	0.44

PPM: patient–prosthesis mismatch; BMI: body mass index; BSA: body surface area; NYHA: New York Heart Association; SEV: self-expanding valve; BEV: balloon-expanding valve. * In its data privacy impact assessment, transferred to the CNIL as the basis for the authorization of the study, the Société Français de Cardiologie established that, to ensure proper anonymization, no result will be provided when they concern a population under 11 subjects. This 11-subject threshold for the publication of results is commonly used and can be found in documents such as the external guidance on the implementation of the European Medicines Agency policy on the publication of clinical data for medicinal products for human use (EMA/90915/2016, Version 1.4).

**Table 2:** Baseline and postimplantation characteristics (PVL cohort).

Variable	All Patients	PVL ≥ 2 (*n* = 4056)	PVL < 2 (*n* = 16,822)	*p*
Duration of follow-up (days since the date of procedure)	781 (356–1341)	803 (350–1351)	777 (357–1339)	0.95
Demography				
Age (years)	84 (80–87)	84 (81–88)	84 (80–87)	<0.001
Female	10,313 (49%)	2034 (50%)	8531 (51%)	0.53
Male	10,565 (51%)	2022 (50%)	8291 (49%)	
BMI	25.7 (23–29)	25.2 (22.5–28.4)	25.8 (23.1–29.1)	<0.001
Body surface (m^2^)	1.79 (1.63–1.93)	1.77 (1.62–1.91)	1.79 (1.63–1.94)	<0.001
Indicators at inclusion				
NYHA III/IV	12,126 (63%)	2295 (61%)	9831 (63%)	0.02
Euroscore 2	4 (2.39–6.86)	3.75 (2.3–6)	4 (2.4–7)	<0.001
Logistic Euroscore	13.57 (8.97–21.71)	13.8 (9–21.56)	13.57 (8.9–21.82)	0.36
History and comorbidities				
Dyslipidemia	6207 (30%)	1132 (28%)	5075 (30%)	0.01
Hypertension	15,425 (74%)	2927 (72%)	12,498 (74%)	0.01
Class IV angina	611 (3%)	104 (3%)	507 (3%)	0.24
Coronary angioplasty	5802 (31%)	1014 (29%)	4788 (32%)	<0.001
Coronary bypass	1865 (9%)	317 (8%)	1548 (9%)	<0.001
Diabetes	5126 (25%)	850 (21%)	4276 (26%)	<0.001
Myocardial Infarction < 90 days	284 (2%)	50 (1%)	234 (2%)	0.64
History of stroke	2288 (11%)	436 (11%)	1852 (11%)	0.6
Chronic renal failure	8874 (48%)	1586 (46%)	7288 (49%)	<0.001
Dialysis	385 (2%)	68 (2%)	317 (2%)	
Creatinine (µmol/L)	92 (74–119)	92 (74–119)	92 (74–118)	>0.99
Chronic obstructive pulmonary disease	3668 (18%)	685 (17%)	2983 (18%)	0.18
Pacemaker	2455 (13%)	474 (14%)	1981 (13%)	0.58
Peripheral arterial disease	4700 (25%)	846 (23%)	3854 (25%)	0.05
Preimplantation examination				
Valve surface (cm^2^)	0.7 (0.57–0.8)	0.7 (0.56–0.8)	0.7 (0.58–0.8)	0.07
Mean gradient (mmHg)	47 (40–57)	48 (40–60)	46 (39–56)	<0.001
Ejection fraction (%)	60 (49–65)	60 (48–65)	60 (50–65)	0.27
Annulus diameter (mm)	24 (22–26)	24 (22–26)	24 (22–26)	0.06
Severe pulmonary hypertension (>60 mmHg)	1807 (11%)	335 (10%)	1472 (11%)	0.17
Aortic regurgitation ≥ 2	3511 (20%)	927 (27%)	2584 (19%)	<0.001
Mitral regurgitation ≥ 2	4138 (24%)	1003 (29%)	3135 (23%)	<0.001
Coronary stenosis (>50%)	7995 (41%)	1506 (40%)	6489 (41%)	0.19
Procedure				
Programmed predilatation	4166 (28%)	814 (29%)	3352 (28%)	0.65
Number of valves implanted > 1	286 (1%)	83 (2%)	203 (1%)	<0.001
Access (iliofemoral)	18,184 (87%)	3581 (89%)	14,603 (87%)	0.01
Valve type				<0.001
SEV	8583 (41%)	2148 (53%)	6435 (38%)	
Corevalve and Corevalve Evolut	3182 (16%)	885 (20%)	2526 (16%)	
Corevalve Evolut Pro	1227 (6%)	269 (7%)	958 (6%)	
Corevalve Evolut R	4174 (20%)	994 (25%)	3180 (19%)	
BEV	12,295 (59%)	1908 (47%)	10,387 (62%)	
Sapien	1923 (9%)	321 (8%)	1602 (10%)	
Sapien 3	8889 (43%)	1240 (31%)	7649 (45%)	
Sapien XT	1483 (7%)	347 (9%)	1136 (7%)	
Diameter of the prosthesis (mm)	26 (23–29)	26 (23–29)	26 (23–29)	<0.001
Postimplantation examination				
Ejection fraction (%)	60 (50–65)	60 (50–65)	60 (50–65)	0.25
Mean gradient (mmHg)	9.7 (7–13)	10 (7–13)	9.25 (7–13)	<0.001
Aortic valve area (cm^2^)	1.8 (1.5–2.1)	1.8 (1.5–2.1)	1.79 (1.5–2.1)	0.78
Indexed aortic valve area (cm^2^/m^2^)	1 (0.83–1.21)	1.01 (0.84–1.23)	1 (0.82–1.21)	0.05
Aortic paravalvular leak ≥ 2	2982 (15%)	2982 (80%)	0 (0%)	
Mitral regurgitation ≥ 2	3047 (18%)	891 (27%)	2156 (16%)	<0.001
Severe pulmonary hypertension (>60 mmHg)	841 (6%)	233 (8%)	608 (5%)	<0.001
Events during hospitalization				
Pacemaker	2888 (14%)	601 (15%)	2287 (14%)	0.
Infection	722 (4%)	144 (4%)	578 (3%)	0.8
ST+ infarction	25 (0%)	<11 (<0.3%)	≥11 (<1%)	0.41
Stroke	324 (2%)	74 (2%)	250 (2%)	0.15
Major bleeding	1240 (6%)	255 (7%)	985 (6%)	0.35
Death	298 (1%)	90 (2%)	208 (1%)	<0.001
Duration of index hospitalization (days)	8 (6–13)	9 (6–14)	8 (6–13)	<0.001
Follow-up data at D30				
Mismatch	1602 (24%)	376 (23%)	1226 (24%)	0.39
Moderate	1289 (19%)	309 (19%)	980 (19%)	0.43
Severe	313 (5%)	67 (4%)	246 (5%)	

PPM: patient–prosthesis mismatch; BMI: body mass index; BSA: body surface area; NYHA: New York Heart Association; SEV: self-expanding valve; BEV: balloon-expanding valve.

**Table 3:** Multivariate adjustment of predictors of PPM.

	Moderate-to-Severe PPM			
Variable	OR	IC 95 Lower	IC 95 Upper	*p*-Value
BMI (per 1 unit increase)	0.958	0.947	0.968	<0.001
Age (per 1 year increase)	0.992	0.987	0.997	0.003
BSA (per 1 m^2^ increase)	8.881	6.718	11.742	<0.001
Baseline ejection fraction (per 1% increase)	0.985	0.982	0.988	<0.001
Diameter size of TAVI valve (per 1 mm increase)	0.848	0.832	0.864	<0.001

BMI: body mass index; BSA: body surface area; PPM: patient–prosthesis mismatch; OR: odds ratio; IC: confidence interval.

**Table 4:** Multivariate adjustment of predictors of PVL.

Variable	OR	IC 95 Lower	IC 95 Upper	*p*-Value
BMI (per 1 unit increase)	0.962	0.951	0.973	<0.001
Body Surface	1.357	1.038	1.774	0.03
Baseline mean gradient (per 1 mmHg increase)	1.009	1.007	1.011	<0.001
Baseline ejection fraction (per 1 mmHg increase)	0.995	0.992	0.998	<0.001
Diameter size of TAVI valve (per 1 mm increase)	0.972	0.959	0.986	<0.001
Type of valve (BEV vs. SEV)	0.509	0.469	0.552	<0.001

BMI: body mass index; SEV: self-expanding valve; PVL: paravalvular leak; OR: odds ratio; IC: confidence interval.

**Table 5:** Multivariate adjustment of predictors of all-cause mortality in PPM cohort.

	Moderate-to-Severe PPM			
Variable	Hazard Ratio	IC 95 Lower	IC 95 Upper	*p*-Value
BMI (per 1 unit increase)	0.992	0.986	0.998	0.009
Age (per 1 year increase)	1.008	1.003	1.012	0.001
NYHA 3/4 (vs. 1/2)	1.225	1.150	1.305	<0.001
Euroscore (per 1% increase)	1.009	1.006	1.011	<0.001
COPD	1.189	1.110	1.273	<0.001
Baseline mean gradient (per 1 mmHg increase)	0.991	0.989	0.993	<0.001
Diameter size of TAVI valve (per 1 mm increase)	1.021	1.009	1.034	0.001
Major bleeding	1.229	1.100	1.374	<0.001
Hospital stay duration (per 1 day increase)	1.020	1.017	1.023	<0.001

BMI: body mass index; COPD: chronic obstructive pulmonary disease; PPM: patient–prosthesis mismatch; OR: odds ratio; IC: confidence interval.

**Table 6:** Multivariate adjustment of predictors of all-cause mortality in PVL cohort.

	PVL ≥ 2			
Variable	Hazard Ratio	IC 95 Lower	IC 95 Upper	*p*-Value
PVL ≥ 2	1.159	1.087	1.237	<0.001
Age (per 1 year increase)	1.012	1.008	1.017	<0.001
BMI (per 1 unit increase)	0.991	0.985	0.996	0.001
NYHA (3/4 vs. 1/2)	1.220	1.149	1.293	<0.001
Euroscore (per 1 unit increase)	1.009	1.007	1.011	<0.001
COPD	1.257	1.178	1.342	<0.001
Baseline mean gradient (per 1 mmHg increase)	0.990	0.988	0.992	<0.001
Diameter of TAVI valve (per 1 mm increase)	1.022	1.010	1.033	<0.001
Major bleeding	1.178	1.068	1.300	0.001
Hospital stay duration (per 1 day increase)	1.018	1.016	1.021	<0.001

BMI: body mass index; COPD: chronic obstructive pulmonary disease; PVL: paravalvular leak; IC: confidence interval.

## Supplementary Materials

The following supporting information can be downloaded at: https://www.mdpi.com/article/10.3390/jcm11206117/s1, Table S1: International Classification of Diseases (ICD-10), medical procedures (national nomenclature, Classification Commune des Actes Medicaux) and medications (ATC) codes used to identify characteristics and outcomes in this study.
