# Supplementary material for: Long-Term Prognosis Value of Paravalvular Leak and Patient–Prosthesis Mismatch following Transcatheter Aortic Valve Implantation: Insight from the France-TAVI Registry
Source: J Clin Med. 2022 Oct 17;11(20):6117. doi: 10.3390/jcm11206117 (PMC9604905; doi:10.3390/jcm11206117)
Supplement: Supplementary file 1 [file jcm-11-06117-s001.zip › jcm-1923904-supplementary.pdf]

Journal of
Clinical Medicine

MDPI

Supplementary Materials

Long-Term Prognosis Value of Paravalvular Leak and Patient–Prosthesis Mismatch Following Transcatheter Aortic Valve Implantation: Insight from the France-TAVI Registry

Supplemental Table S1. International Classification of Diseases (ICD-10), medical procedures (national nomenclature, Classification Commune des Actes Medicaux) and medications (ATC) codes used to identify characteristics and outcomes in this study.

Variable Category	Type	Codes
**Inclusion criteria**		
Valve-in-valve	CCAM	DBKA009; DBMA009; DBMA010; DBMA006; DBKA011; DBKA003; DBKA001; DBMA004; DBPA007; DBMA001; DBMA015; DGKA014
TAVI-in-TAVI	CCAM	DBLF001; DBLA004
**Comorbidities**		
Hypertension	ICD-10	I10x; I11x; I13x; I674
	ATC	C02; C08
Dyslipidemia	ICD-10	E780; E781; E782; E783; E784; E785
**Early and late clinical outcome (occurring after the index hospitalization)**		
Cardiac device implantation (pacemaker, CRT-P, CRT-D, DAI)	CCAM	DELF005; DELF903; DELF010; DELF007; DELA001; DELA003; DELF223; DELF086; DELF013; DELA004; DELF900; DELF020; DELF014; DELF901; DELF904; DELF905; DELF012; DELF015; DELF001; DELF902;
Stroke	ICD-10	I63x; I62x; I61x; I64x; G8100; G45x
Arrhythmia	ICD-10	I48x; I49x; I45x; I471; I472
Aortic valve reintervention (aortic valve replacement, redo TAVI, balloon aortic valve valvuloplasty, PVL closure)	ICD-10	T820 ; T825 ; T826 ; T827 ; T828 ; T829
	CCAM	DBKA006 ; DBKA009 ; DBMA009: DBMA010; DBMA006; DBKA011; DBKA003 ; DBKA001 ; DBLF001 ; DBLA004 ; DBAF001 ; DBSF001 ; DBMA004 ; DBPA007 ; DBKA003 ; DBMA001 ; DBMA015 ; DGKA014
Heart failure hospitalization	ICD-10	I50x ; R570
